# Toward the Development of an On-Chip Acoustic Focusing Fluorescence Lifetime Flow Cytometer

**DOI:** 10.3389/fphy.2021.647985

**Published:** 2021-05-14

**Authors:** Jesus Sambrano, Felicia Rodriguez, John Martin, Jessica P. Houston

**Affiliations:** 1 Department of Chemical and Materials Engineering, New Mexico State University, Las Cruces, NM, United States,; 2 Tiber Plasma Diagnostics, Las Cruces, NM, United States

**Keywords:** flow cytometry, FRET, time-resolved flow cytometry, fluorescence lifetime, acoustofluidic

## Abstract

Conventional flow cytometry is a valuable quantitative tool. Flow cytometers reveal physical and biochemical information from cells at a high throughput, which is quite valuable for many biomedical, biological, and diagnostic research fields. Flow cytometers range in complexity and typically provide multiparametric data for the user at rates of up to 50,000 cells measured per second. Cytometry systems are configured such that fluorescence or scattered light signals are collected per-cell, and the integrated optical signal at a given wavelength range indicates a particular cellular feature such as phenotype or morphology. When the timing of the optical signal is measured, the cytometry system becomes “time-resolved.” Time-resolved flow cytometry (TRFC) instruments can detect fluorescence decay kinetics, and such measurements are consequential for Förster Resonance Energy Transfer (FRET) studies, multiplexing, and metabolic mapping, to name a few. TRFC systems capture fluorescence lifetimes at rates of thousands of cells per-second, however the approach is challenged at this throughput by terminal cellular velocities. High flow rates limit the total number of photons integrated per-cell, reducing the reliability of the average lifetime as a cytometric parameter. In this contribution, we examine an innovative approach to address this signal-to-noise issue. The technology merges time-resolved hardware with microfluidics and acoustics. We present an “acoustofluidic” time-resolved flow cytometer so that cellular velocities can be adjusted on the fly with a standing acoustic wave (SAW). Our work shows that acoustic control can be combined with time-resolved features to appropriately balance the throughput with the optical signals necessary for lifetime data.

## INTRODUCTION

Flow cytometry is a robust statistical apparatus common to many research fields as well as the medical industry. Cytometry methods involve analyzing individual cells with the aid of laser excitation light, which excites fluorophores conjugated to the cell of interest. Specialty photo- detectors measure the intensity of the emitted fluorescence signal or excitation light that is scattered by the cell. This high-throughput technique is also used to separate subpopulations of a heterogeneous population of cells based on the fluorescence signal measured. The excitation light that is scattered by the cell is directionally measured (forward scatter and side scatter) and also provides information about the cell—generally morphological changes such as size, granularity, etc. [1].

Recent advances in lasers and data acquisition systems have led to the ability to measure, cytometrically, an additional fluorescence parameter, this being the fluorescence lifetime [2]. Uniquely designed cytometry approaches that extract fluorescence lifetimes are known as time-resolved flow cytometry (TRFC) systems. The fluorescence lifetime parameter is independent of the fluorescence intensity and can provide additional multiplexing capabilities [3–5]. For example, TRFC was recently employed in an application in which the endogenous fluorophore, NAD(P)H, was measured to identify metabolic changes during apoptosis since it is well-known that this metabolite undergoes fluorescence lifetime shifts during enzymatic reactions important for energy generation [6]. To obtain the fluorescence lifetime of a fluorescent compound, a modulated laser excites the fluorophore on or within the cell. This modulated excitation source leads to a fluorescence signal modulated at the same frequency and a similarly modulated scattered light signal [2, 7, 8]. Due to the fluorescence decay, a phase difference between the scattered laser light and the emitted fluorescence results. The fluorescence lifetime is thus calculated from the difference in these phases.

One other significant area of development in flow cytometry is throughput and synergism with imaging and on-chip detection. Many advancements have been made with cytometers that employ micron-sized channels to control and manipulate cells. Microfluidic devices have an abundance of advantages over disadvantages. When small volumes and quantities are used, then sample analysis and sorting becomes facile. Additionally, microfluidic systems are favored because miniaturization leads to reduce operational costs. Biomedicine has been most significantly impacted by microfluidic approaches [9, 10] that also contain microelectromechanical systems (MEMS), or more commonly, “lab on a chip” [11] devices. MEMS provide a way to perform analytical measurements based on passive and proactive interrogation. These lab-on-a-chip systems thus enable cells to be aligned, stretched, deflected, labeled, treated, excited, lysed, sequenced, and more. In other words, the control and manipulation of cells is quite impressive with microfluidics, and the clever chip-based systems found today are not only versatile but also portable, low-cost, and massively parallelized. In general, the MEMS systems that incorporate optical signals for cell interrogation involve careful control of the cell as it is moved through small channels and across a focused laser beam. For single-cell analyses, cells must be aligned and ideally move in single file. The alignment can be accomplished by dielectrophoretic focusing [12], inertial focusing [13], magnetophoresis [14], or acoustophoresis [15], to name a few of the most widely used approaches. The end goal for such systems is to achieve high spatiotemporal resolution resulting in a crisp image of a single cell, or an ample optical signal that can be used to make interpretations about the cell (i.e., phenotype, genotype, etc.).

Herein we combine both emerging areas of flow cytometry: time-resolved measurements and microfluidics. These technologies are merged and discussed in this contribution for the purpose of achieving higher fluorescence lifetime resolution and to enable the detection of low-level optical signals from cells. Our objective is to adapt TRFC onto a microfluidic platform for control with acoustics to achieve the necessary fluorescence sensitivity and high temporal resolution (~100 s of picoseconds) during cell counting and eventually sorting. Interestingly, there have been very few TRFC systems designed onto microfluidic chips that measure real-time fluorescence lifetimes. The fluorescence lifetime is challenging to read-out during cell measurements because the timing of decay must be calculated with on-chip digital signal processing using frequency-domain analyses. Although in past work we implemented the fluorescence lifetime as a sorting parameter on a traditional cytometer, we found it challenging to sample the optical signals at an adequate level. A higher signal-to-noise is necessary for higher timing resolution. Unfortunately the tradeoff remains; cells can either be moved through a laser at a high throughput (~1–10 μs), or slowed down so that the amount of time that photons can be collected is increased (100 μs−100 ms) for a robust lifetime read-out.

In this contribution we adopt microfluidics in order to evaluate how a slower flow rate might improve our ability to perform TRFC. We thus apply standing acoustic waves (SAW) for cell movement and alignment. This acoustophoretic approach works synergistically with quiescent and laminar flow regimes. Particles/cells are transported by a laminar flow, and their motion is controlled dependent on a set of cellular physical properties such as particle compressibility, particle size, and density. With SAW, piezoceramic actuators composed of perovskite crystals are mechanically excited to propagate an ultrasonic sound wave that permeates through a matching layer, meeting, and reflecting off the reflecting layer to produce a SAW with respect to the microchannel geometry, composite material of the microchannel, and the speed of sound in the fluid. With more control over photon collection and cell movement, we plan to make it possible to calculate more than one fluorescence lifetime per cell (i.e., multiple decay kinetic acquisition), and address outstanding questions in the fluorescence lifetime field. The following report discusses work toward this goal.

## METHODS AND MATERIALS

### System Overview

**FIGURE 1 |:**
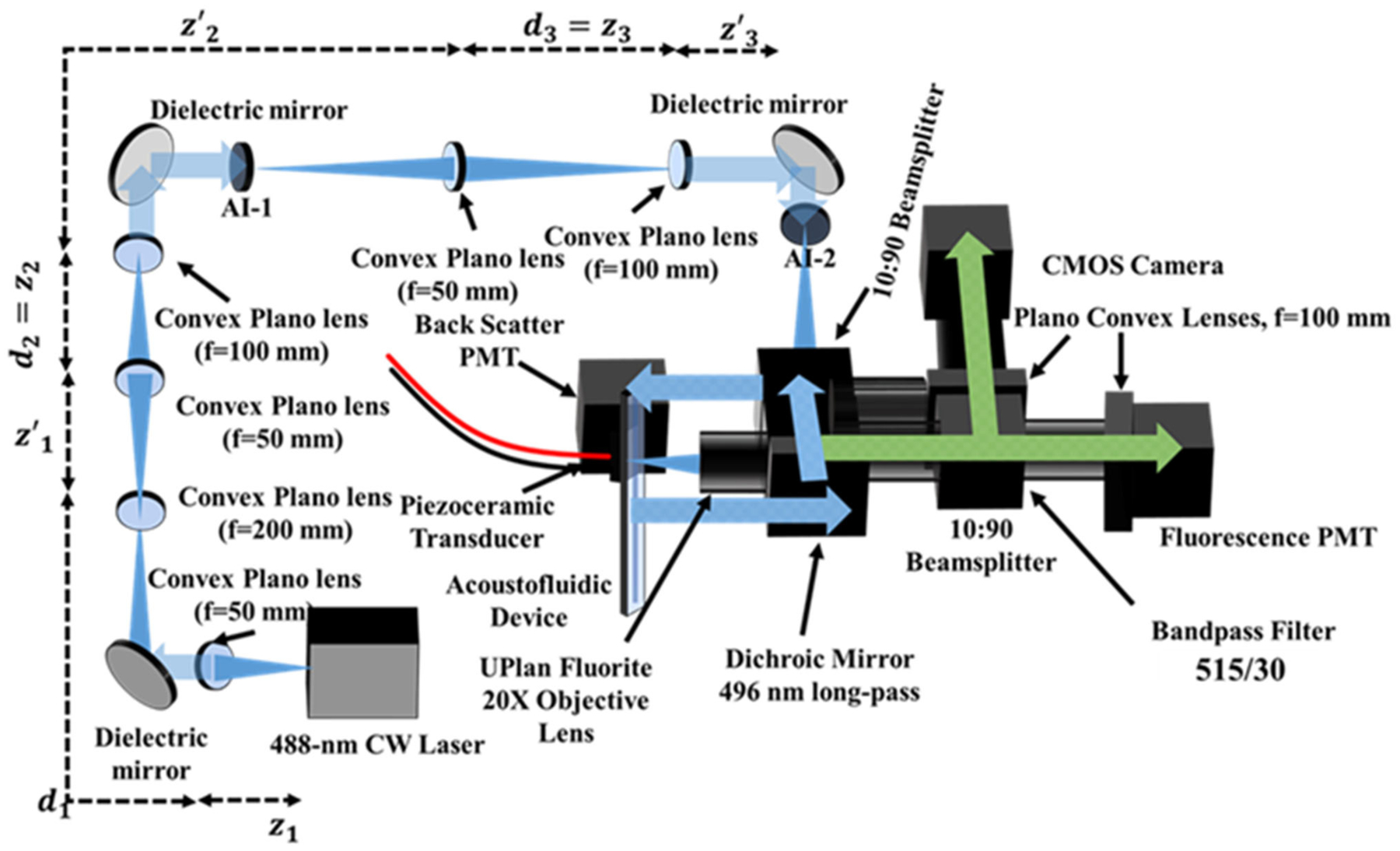
Schematic representation of the TRAFFC system. The system is an epifluorescence-like arrangement where emission light is reflected and collected at 0° (fluorescence and imaging CMOS camera). Light scatter is collected through low angle scattering through the dichroic beam splitter and plate beam splitter. To achieve a laser spot size commonly found in commercial cytometers, the laser beam is expanded three times before being focused down onto the sample plane on the acoustofluidic device. Optical aberrations are cleaned with adjustable irises (AI).

The time-resolved acoustofluidic flow cytometer (TRAFFC) described herein (see Figure 1) was designed to be modular. Each component was procured, assembled, and optimized to fit onto a standard 2 × 2 optical breadboard. The components include the chip, optics, detectors, electronics, and data system described herein.

BennuBio, Inc (Albuquerque, NM) kindly gifted us the acoustic chip, which consisted of an aluminum core (125 × 12 mm) with a 500 μm wide microchannel. The aluminum core is bonded between two borosilicate slides (125 × 12 mm) which create the matching and reflecting layer for acoustic focusing. The inlet and outlets contain luers (IDEX Corp., Northbrook, IL) epoxied onto respective outlets. On the reverse side of the device is a piezoceramic (PZT, lead zirconate titanate) transducer (30 × 30 mm). Two leads extend outward from the PZT and are connected to a power amplifier (Electronics and Innovation, LTD, Rochester, NY) and arbitrary function generator (Tektronix, Beaverton, OR), respectively. Sample is introduced to the acoustofluidic device using a Chemyx Fusion 200 syringe pump (Stafford, TX). When initially optimizing the system and establishing a fluorescence baseline, a flow rate of 50–100 μL/min was used. As fluorescence detection was established, flow rates were varied between 10 and 500 μL/min. This work used flow rates of 250 μL/min.

The TRAFFC optical system was developed with a single laser excitation source, a 2-channel detector arrangement (fluorescence and light scatter), and a CCD camera for imaging. The excitation source was a blue diode laser (OBIS LX 488 nm, Coherent Inc., Santa Clara, CA) with a maximum optical power output of 150 mW. To assure minimal reflection from the device channel walls, the laser spot was minimized to a Gaussian-like profile with a diameter of ~180 μm through a series of three-lens expansion systems. The first expansion set of lenses had focal lengths of 50 and 200 mm, respectively. The second and third set of lenses consisted of lenses with focal lengths of 50 and 100 mm as diagrammed in Figure 1. The laser reflected off a dichroic beam splitter (495LP, Semrock, Rochester, NY) and was directed to the acoustofluidic device. For steady-state fluorescence measurements, the laser operated at 50 mW in continuous wave mode. For acquisition of fluorescence decay kinetics (fluorescence lifetime), the laser operated in digital modulation mode and was modulated at a sinusoidal radio frequency of 195 kHz by an arbitrary function generator. Fluorescence was collected by an objective (LMPLFLN 20X, NA=0.4, Olympus, Waltham, MA), passed through a bandpass filter (515/30 nm, Semrock, Rochester, NY), and was split into two image components (10:90 plate beam splitter, Thorlabs, Newton, NJ). The emission signal was captured by a photomultiplier tube (PMT, Hamamatsu model type R928) and a CMOS camera (ORCA-Flash4.0 V3 CMOS Hamamatsu Photonics, San Jose, CA). Light was focused onto the camera sensor and PMT window through respective Plano convex lenses (*f* = 100 mm, Thorlabs, Newton, NJ). A PMT for optical backscatter was positioned after a second 10:90 plate beam splitter. The fluorescence PMT voltage was set at an offset of −480V, and the exposure time for the CMOS camera was 32 ms (~40 frames per second). The camera was operated using HCImage Live software suite (Hamamatsu Corporation, Sewickley, PA) with a Dell OptiPlex computer (32 GB of RAM, Round Rock, TX. Data were collected using a custom data acquisition system with a sampling rate of up to 50 megasamples per second (50 MSPS); data transfer was through a FireWire 400 cable to a FireWire PCIe adapter.

### Testing Samples

Fluorescent microspheres as well as fluorescently labeled cells were used to evaluate the performance of the preliminary TRAFFC system. Standard flow cytometry microspheres with known fluorescence lifetimes included FlowCheck^®^ fluorospheres (Beckman Coulter, Indianapolis, IN) and Yellow-Green (YG) fluorospheres (Polysciences Inc., Warrington, PA). All microspheres and cells were prepared at a concentration of ~1.0 × 10^6^ particles/mL. The cells used for testing included a MCF-7 breast cancer line. Cells were cultured in 5 mL DMEM containing 10% by volume fetal bovine serum, 1 mM of sodium pyruvate, and 2 mM of L-glutamine to make a final concentration of 1× for the latter two components. The cells, cultured in a 37°C, 5% (v/v) CO_2_ environment, were harvested when concentrations reached ~1.5 × 10 cells/mL. Additional cell preparation for TRAFFC measurements included fixation and labeling with the fluorescence dye, propidium iodide (PI). The protocol included removing cells from culture and centrifuging at 350 × G for 5 min. Cells were then washed twice with DPBS, gently vortexed and fixed with 3 mL of 70% ethanol. Fixation included storing cells for ~1 h in a −20°C freezer. Cells were then centrifuged at 1,150 RPM for 5 min and re-suspended in 7 mL of 50 μg/mL RNase. Fluorescence labeling included adding 35 uL of propidium iodide at 37°C for 30 min. The final working concentration for the MCF-7 cells was verified by a hemocytometer and the Countess II Automated Cell Counter (Thermo Fisher Scientific, Waltham, MA,). Fluorescent labeling for MCF-7 cells were also checked prior to experiments with an ECHO Revolve Microscope (ECHO, San Diego, CA.) with a 10X objective in both upright mode and under fluorescence inverted mode (data not included).

## RESULTS

### General Fluorescence Measurements

**FIGURE 2 |:**
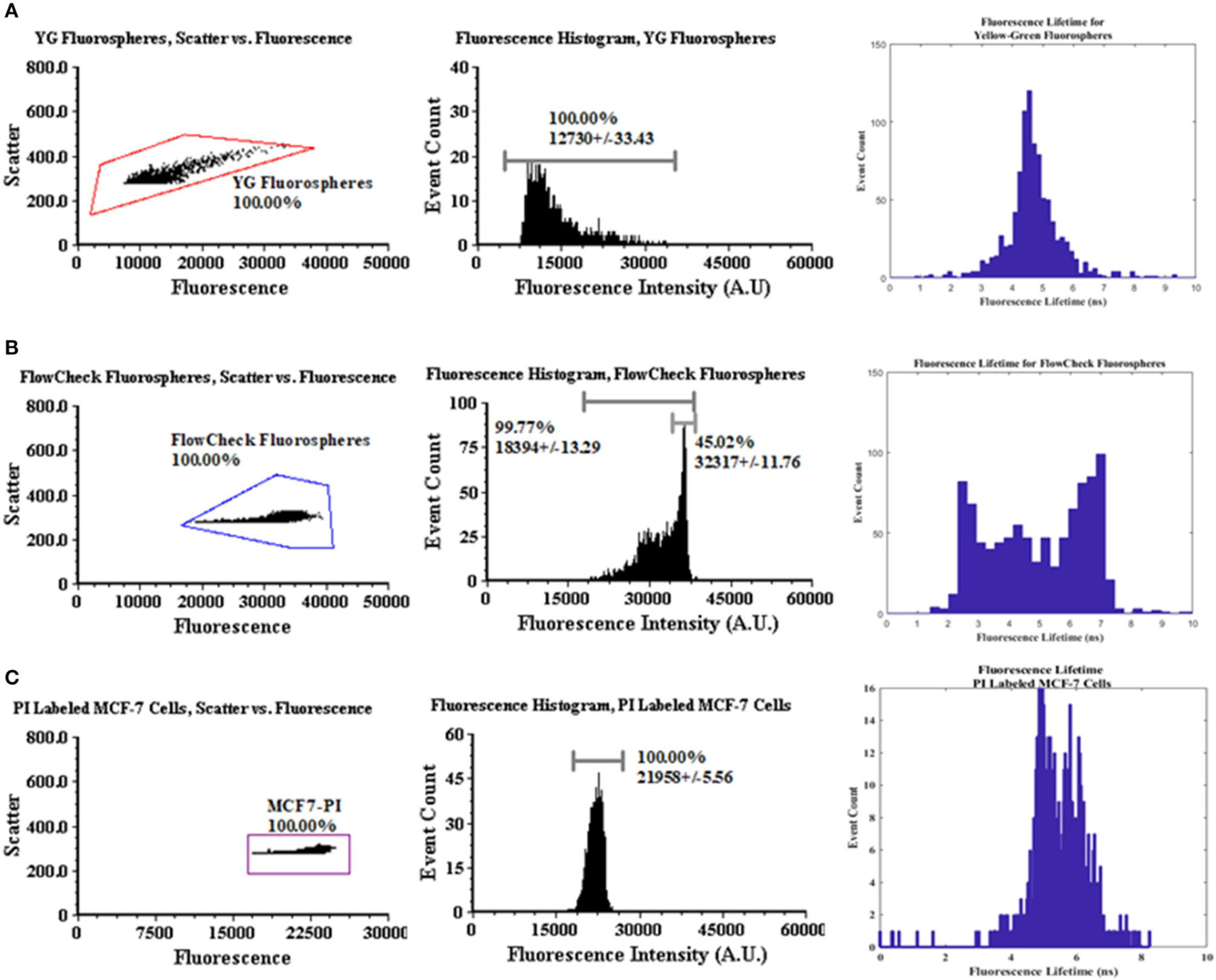
Fluorescence and reference waveforms plotted, and resultant frequency spectrums analyzed offline in MATLAB for **(A)** YG fluorospheres, **(B)** FlowCheck^®^ fluorospheres, and **(C)** PI-labeled MCF-7 cells.

The preliminary tests performed on the TRAFFC system included fluorescence measurements detected from both fluorescence microspheres and the PI-labeled cells. Future work will optimize the of capture backscattered light, which is important for cell characterization and robust fluorescence lifetime calculations and is discussed later. The fluorescence signals from the PMT were amplified with a transimpedance amplifier (TIA, DC-100, ARI Corp.) with a low noise 60 dB gain and overall low voltage signal contribution of 1mV, peak-to-peak. The fluorescence signals after amplification were observed using an oscilloscope as well as the custom DAQ system described previously. Figure 2 is a graph showing examples of the fluorescence data collected. The signals have a “modulated pulse” shape, similar to standard digital cytometry measurements we have performed previously [8]. The signals expectedly follow an increase in fluorescence and decrease as an acoustically focused microsphere and/or cell enters the laser excitation site and rapidly exits. These waveforms after collection can be compiled and stored as comma separated value files (.csv) for off-line analysis. Accordingly, MATLAB (Natick, MA) was used to read and plot the waveforms for further investigation. As can be seen by Figure 2, the pulse width is wide, which indicates a mean flow velocity (0.16670.1·m s ^−1^) and mean differential pressure of ~11 PSI that fosters a longer dwell time in the beam. An item to note is how the outlet of sample to waste may affect these dwell times. The outlet waste tube is submerged in fluid at all times to negate backpressure “shockwaves” up through the outlet tube that would be incurred by break-off droplets. However, by keeping the outlet tube submerged, it is possible that changes in differential pressure occur and may affect sample velocity and signal duration (pulse widths).

In addition, the TRAFFC system uses standing acoustic wave (SAW) focusing, an alternative to sheath-driven hydrodynamic focusing. A more in-depth analysis of acoustic focusing is discussed later. Briefly, SAWs generated a pressure node across the center cross-section of the microchannel. A modest flow rate was used to assure particles/cells had sufficient time to be captured by the SAW. Finally, to enable optimized acoustic focusing and, maximum dwell time within the detection aperture, particle focusing was initiated 3 mm downstream from the detection aperture. Figure 2 includes the modulated reference signal plotted with the fluorescence pulse to demonstrate DAQ capture of both for fluorescence lifetime measurements. Finally, Figure 2 includes the frequency spectrum results of the Fourier analysis, which is one step toward calculating the fluorescence lifetime (further described below). Transit times are also plotted against fluorescence intensities.

### Intensity and Lifetime Measurements

**FIGURE 3 |:**
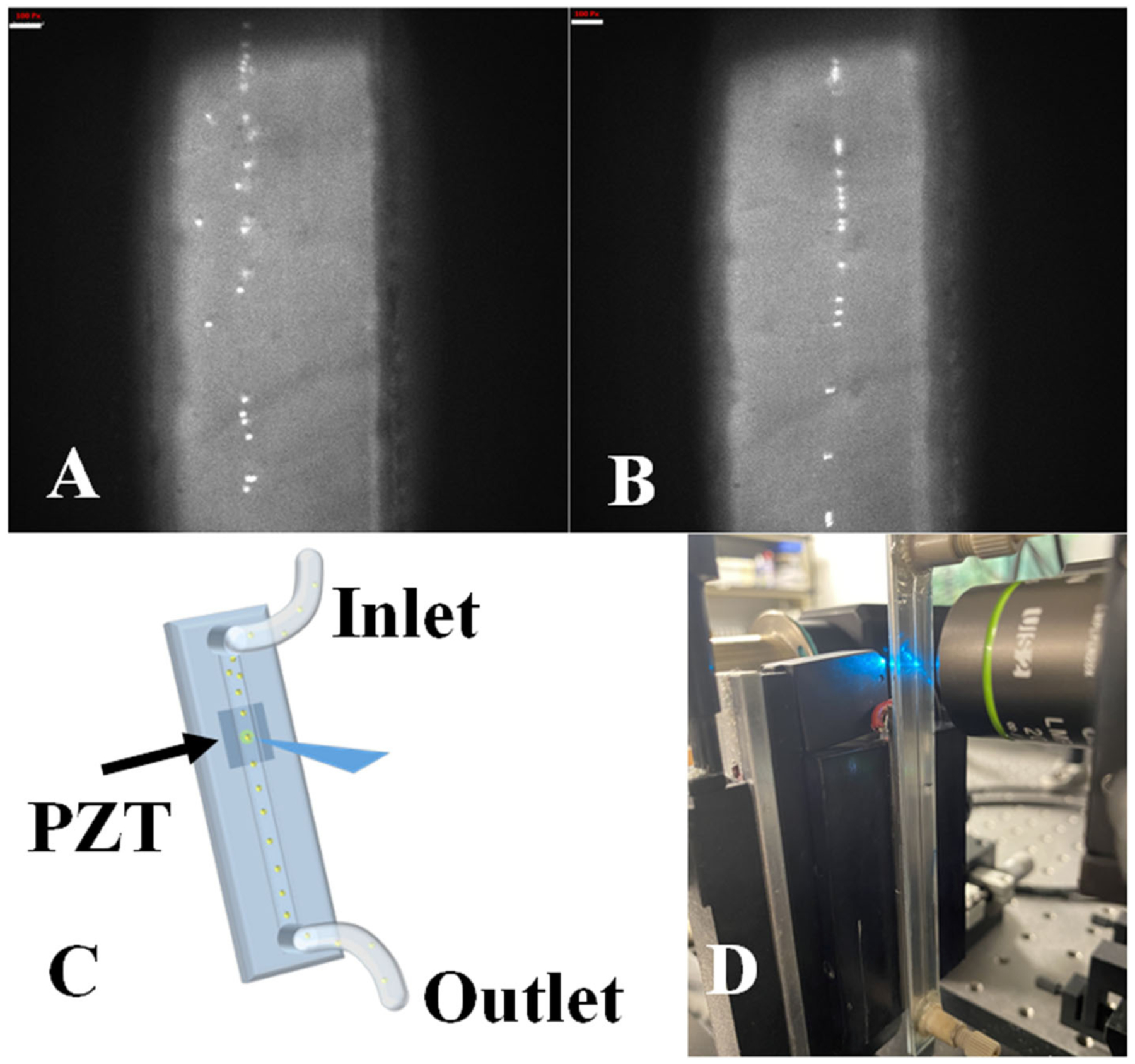
Dot plot, intensity, and lifetime histogram data for fluorospheres and MCF-7 cells. **(A)** A polygon gate was applied to the entire dot plot population for YG fluorospheres. The mean intensity was recorded at 12730 A.U. with a CV of 33.43%. The corrected mean lifetime was recorded as 5.07 ± 1.66 ns from DAQ acquisition. **(B)** The same method was applied for FlowCheck^®^ fluorospheres with respect to the dot plot gating. Mean intensity value was measured at 18394 A.U. with a CV of 13.29%. The mean corrected lifetime was measured at 5.07 ± 1.66 ns with visible bi-modal populations. **(C)** Propidium-labeled MCF-7 cells were acquired for up to 1,000 events. Mean fluorescence intensity was measured at 21958 A.U. with a CV of 5.56%. Mean corrected lifetime was measured at 5.73 ± 0.76 ns. **(D)** The acoustofluidic device used in the modular TRAFFC system.

Figure 3 provides a statistical summary of the fluorescence intensity and lifetime measured of the fluorescence microspheres and fluorescently labeled cells. Microsphere data collection was repeated 6 times after calibration. To calibrate the lifetime measurements, we used YG fluorospheres as a calibration reference, which have a known lifetime value ~3 ns. Thus, we calculated the anticipated phase shift value (Δϕ_expected_) to adjust the signals prior to measuring other microspheres and cells. For the calibration analysis, ~3,000 events were collected. Data were analyzed offline in MATLAB (as seen in Figure 2). A Discrete Fourier Transform (DFT) was applied to the fluorescence and reference waveforms. The frequency spectrum revealed the DC and modulation frequencies for each respective waveform. Phase angles were extracted for each waveform and the phase shift value was calculated (ϕ_FL_ − ϕ_Reference_ = Δ ϕ_calculated_). Phase correction was then made and applied to all experiments for the remainder of the measurements made.

Also After calibration, FlowCheck^®^ fluorospheres were measured with the TRAFFC system (~3,000 events for *n* = 6 experiments). Data included the mean intensity, event count, and percent coefficient of variation (standard deviation/mean × 100). The FlowCheck^®^ fluorospheres displayed a bright intensity at a mean intensity of 32317 A.U. with a CV of +/−11.76%. This is in comparison to YG fluorospheres (mean fluorescence intensity = 12730 a.u and cv = 33.43%). Finally, MCF-7 breast cancer cells were evaluated (*n* = 6 trials, and ~1,000 events). The mean fluorescence intensity from the labeled cells was measured to be 21958 a.u. with a cv of 5.56%.

Fluorescence lifetime analyses were performed on FlowCheck^®^ Fluorospheres, YG fluorospheres and PI-labeled MCF-7 cells. We collected waveforms at a low sampling frequency (195 kHz) to adjust for digitization of long pulse widths. Waveforms were analyzed when collected by both the laboratory oscilloscope as well as a DAQ system. Data acquired by the oscilloscope included only a single particle/cell measurement whereas DAQ-recorded waveforms included up to 1,000 events. Lifetimes calculated from the oscilloscope are provided in Table 1. The FlowCheck^®^ and YG fluorospheres recorded a mean lifetime value of 6.25 ± 1.66 ns and 5.16 ± 1.51 ns, respectively. These values are close to many previous reports for these fluorescence microspheres [4]. DAQ-derived lifetimes were calculated to be 5.07 ± 1.66 ns for FlowCheck^®^ fluorospheres and 3.01 ± 7.63 ns for YG fluorospheres. The PI-labeled MCF-7 cells were measured similiarly and processed for average fluorescence lifetime measurements. The oscilloscope-derived mean lifetime for the PI when intercalated into a cell was calculated to be 8.38 ± 2.09 ns. When the PI-labeled cells were processed in bulk after collection by the DAQ, a calculated mean lifetime value was found to be 5.73 ± 0.76 ns. Propidium iodide is known to have an average fluorescence lifetime when intercalated into the DNA of a cell that ranges from 8 to 12 nanoseconds. Our results include a wider lifetime range, which suggests the need to optimize the modulation frequency and make digitization adjustments when longer pulses are collected.

### Acoustic Focusing

Upon evaluating the acoustic focusing component of the system, we found optimal settings necessary for measuring cell and microsphere signals. The TRAFFC system used one-dimensional acoustic focusing as opposed to two-dimensional focusing as found in recent studies by others [16]. One dimensional focusing involves acoustic focusing applied only across the width of the channel as opposed to the width and height of the channel. From here, we calculate the theoretical frequency needed to create a pressure node (SAW) at the center of the microchannel. The equation is as follows:

$$f=\frac{c_{m}}{2D}$$

where c_m_ is a constant and is the speed of sound in water which is 1,480 m s^−1^. The width of the channel for our acoustofluidic device is 0.5 mm. After calculating with these numerical values, we concluded a frequency of 1.48 MHz that is needed to generate a single pressure node for acoustic focusing. In experimental analysis, we found that best focusing for polystyrene beads >5 μm was found at 1.53 MHz with amplitude of −9 dB that yielded power amplified output of 12 V_p−p_. Our one-micron beads required an amplitude of −2.5 dB, leading to a power amplified a 16 V_p−p._ MCF-7 cells required a 1.51 MHz driving frequency but a lower amplitude of −12 dB, netting a voltage of 9 V_p−p._

## DISCUSSION

The data collected from the TRAFFC system described herein are preliminary, and although this modular cytometer requires further optimization, many steps have been achieved to establish lifetime measurements in combination with acoustically-driven flow of samples through a microchip. The TRAFFC system is capable of slower flow velocities and can adequately focus cells and microspheres for measurement. We observed longer integration times compared to conventional TRFC systems we have developed in past work. Importantly, the larger pulse widths, required us to reduce the digitization rate; this limitation will be a focus of our future work. In general, the significance of this preliminary work is that acoustic focusing can be accomplished for lifetime analysis with cells and microspheres, and this is an important step toward control of sample for eventual multi-lifetime analyses and sorting. With the FlowCheck^®^ and YG fluorospheres, as well as PI-labeled cells, we measured bright and reproducible fluorescence intensity and fluorescence lifetime measurements. In the following sections we draw conclusions from this work related to the areas that require significant improvement, which include detection of light scatter, data acquisition, and acoustic control.

## LIGHT SCATTER

The current design we present herein is that of an acoustically focused cytometer without the ability to collect light scattered from cells. Scattered light from the laser excitation source is important in cytometry because it roughly provides morphological and cytoplasmic features synergistically with fluorescence. The scatter parameter may also be of importance for sorting and separation of cells from debris. The TRAFFC system we present is designed to capture optical backscatter, which is currently challenged by de-focused background light. One way to optimize this is to design the TRAFFC system such that the acoustofluidic device is oriented horizontally (angling the device for low angle scattering capture) so as to negate background laser intrusion into the scatter optical channel. While it is possible to fabricate a device with optical waveguides or use of ultrasonic backscatter, we seek to reduce the complexity and costs associated with modular cytometry system.

## DATA ACQUISITION

During the acquisition of fluorescence data we found that the transit time of the cell through the excitation source was indeed slower (relative to traditional cytometry). Yet this slow-down led to long cytometric pulses. The width of the pulse required down sampling of the digitized pulses thus limiting our real-time analysis of the fluorescence lifetime. However, this did not hinder our ability to acquire fluorescence lifetimes in the nanosecond range. Time resolutions and transit time spreads for our DAQ, oscilloscope and PMT are 1 μs, 500 ps, and 1.5 ns, respectively. We found that reducing the sampling rate to 0.195 MHz, and ADC clocks to 1.56 MHz allowed for the capture of the entire fluorescence pulse. Another feature is that the DAQ offers the ability to control modulation cycles that are used to calculate the DFT, which calculates the phase shift needed for lifetime measurements. This is significant to our offline analysis. When we perform a Discrete Fourier Transform (DFT) on a single waveform or in bulk, we can expect the phase extracted from the frequency analysis to be very close to the actual phase value that is unique to the fluorophore. Increased modulation cycles minimizes phase errors. Error rates have been demonstrated in the past to be lower (phase shift errors at or below 0.4°) at modulating frequencies under 10 MHz. This translates to lifetime errors estimated to be around ~0.10 ns. At smaller pulse widths, the increase in cycles would have a minimal effect whereas larger pulse widths see a more dramatic change with respect to error minimization. Although increased pulse widths can decrease the fluorescence lifetime standard deviation, when the modulation frequency is low, this variance is high. That is, at a higher the frequency the number of cycles increases; at the same high frequency a shorter pulse would have a less accurate DFT calculation compared to a longer pulse. Thus, in this work we show that the pulse width can be achieved and with adequate frequencies we should be able to achieve very high-resolution fluorescence lifetimes within tenths of a nanosecond.

## ACOUSTIC FOCUSING

**FIGURE 4 |:**
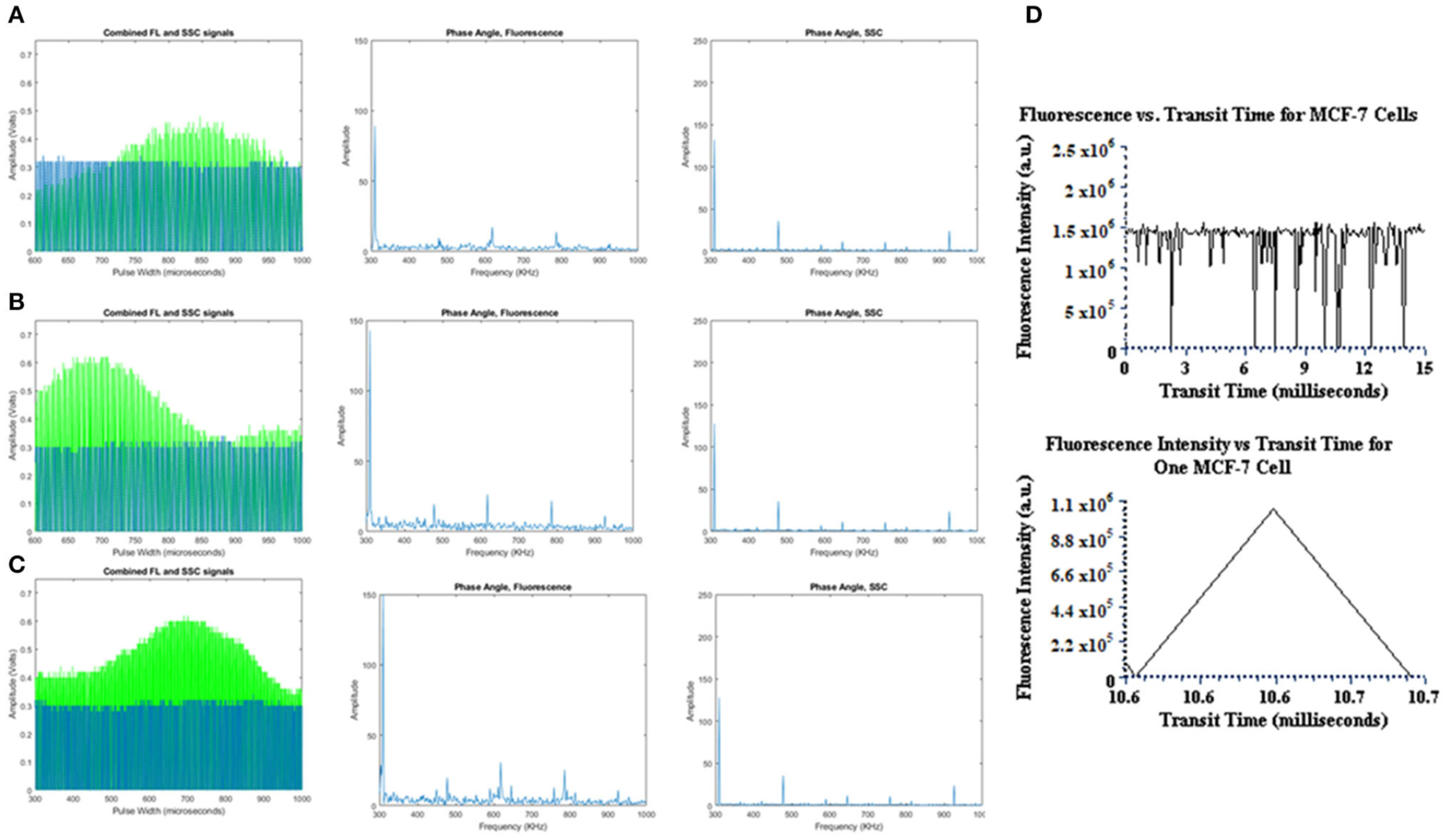
Acoustic focusing performance. **(A)** Unfocused FlowCheck^®^ particles passing through the device at 10 μL/min. White bar equals 100 pixels. **(B)** Acoustic field activated, and particles align to the pressure node in the center of the device’s channel. **(C)** Graphical representation of acoustic focusing in-action. As the particles pass through the PZT generated SAW, alignment takes place along central pressure node. **(D)** Transit time in milliseconds vs. fluorescence intensity plotted for MCF-7 cells (up to 15 milliseconds) and the transit time (~200 μs) for a single MCF-7 cell in milliseconds.

Finally, we determined that while acoustic focusing is possible there is a range of optimal frequencies that will dictate future measurements depending on the cell and sample measured. Upon evaluating the acoustic performance (Figure 4) we found that alignment was possible for particles as small as 3-micron with minor adjustments to acoustic parameters. Larger particle sizes aligned to the pressure nodes at higher flow rates which is also promising for higher-throughput measurements. In the data we present herein (cytometry histograms), cells alignment outperformed microsphere alignment (based on %cv), which is likely due to the density of the cells and the acoustic settings chosen. Owing to the fact that acoustic focusing has been optimized by others, we can draw on knowledge of what frequencies are optimal and identify the theoretical frequencies needed with our specific PZT (i.e., optimum resonant frequencies such as 1 vs. 0.1 kHz steps). These parameters are generally straightforward for optimization of our TRAFFC system.

Overall, this short report presents steps toward the development of a fully functional TRAFFC system. We provide evidence that fluorescence lifetimes can be measured from cells and microspheres with a goal to continue to refine the optofluidic design. We plan to focus on the limitations in the optical measurements as well as real-time data acquisition. Our goal is to use the TRAFFC system for key applications such as FRET, immunophenotyping, lanthanide-labeled vesicle detection, and metabolic mapping. Ultimately this instrument can be transformative for the flow cytometry field at large.

## Figures and Tables

**TABLE 1 |:** Mean fluorescence intensity values, oscilloscope and DAQ calculated fluorescence lifetime values for FlowCheck fluorospheres, yellow-green fluorospheres and PI-labled MCF-7 cells.

Particle	Mean intensity (A.U.)	CV%	Rigol oscilloscope calculated *τ* (ns)	DAQ calculated *τ* (ns)
FlowCheck® fluorospheres	32317	11.76	6.25 **±** 1.66	5.07 ± 1.66
Yellow-green fluorospheres	12730	33.43	5.16 **±** 1.51	3.01 **±** 7.63
PI Labeled MCF-7 Cells	21958	5.56	8.38 **±** 2.09	5.73 ±0.76

## References

[1] Shapiro H. Practical Flow Cytometry. Hoboken: John Wiley & Sons (1995).

[2] Houston JP, Naivar MA, Jenkins P, Freyer JP. Capture of fluorescence decay times by flow cytometry. Curr Protocols Cytometry. (2012) 59:1–21. doi: 10.1002/0471142956.cy0125s59 PMC4240630 25419263

[3] Cao R, Jenkins P, Peria W, Sands B, Naivar M, Brent R, Phasor plotting with frequency-domain flow cytometry. Opt Expr. (2016) 24:14596–607. doi: 10.1364/OE.24.014596 PMC5025209 27410612

[4] Cao R, Pankayatselvan V, Houston JP. Cytometric sorting based on the fluorescence lifetime of spectrally overlapping signals. Opt. Express (2013) 21:14816–31. doi: 10.1364/OE.21.014816 23787669 PMC3726248

[5] Jenkins P, Naivar MA, Houston JP. Toward the measurement of multiple fluorescence lifetimes in flow cytometry: maximizing multi-harmonic content from cells and microsphere. J Biophotonics. (2015) 8:908–17. doi: 10.1002/jbio.201400115 25727072 PMC4869968

[6] Alturkistany F, Nichani K, Houston KD, Houston JP. Fluorescence lifetime shifts of NAD (P) H during apoptosis measured by time-resolved flow cytometry. Cytometry Part A. (2019) 95:70–9. doi: 10.1002/cyto.a.23606 PMC6587805 30369063

[7] Cao R, Naivar MA, Wilder M, Houston JP. Expanding the potential of standard flow cytometry by extracting fluorescence lifetimes from cytometric pulse shifts. Cytometry A. (2014) 85:999–1010. doi: 10.1002/cyto.a.22574 25274073 PMC4257068

[8] Houston JP, Naivar MA, Freyer JP. Digital analysis and sorting of fluorescence lifetime by flow cytometry. J Int Soc Analyt Cytol. (2010) 77:861–72. doi: 10.1002/cyto.a.20930 PMC2930036 20662090

[9] Tabeling P Introduction to Microfluidics. Oxford University Press on Demand (2005).

[10] Whitesides GM. The origins and the future of microfluidics. Nature. (2006) 442:368–73. doi: 10.1038/nature05058 16871203

[11] Verpoorte E, De Rooij NF. Microfluidics meets MEMS. Proc IEEE. (2003) 91:930–53. doi: 10.1109/JPROC.2003.813570

[12] Çetin B, Li D. Dielectrophoresis in microfluidics technology. Electrophoresis. (2011) 32:2410–27. doi: 10.1002/elps.201100167 21922491

[13] Zhou J, Papautsky I. Fundamentals of inertial focusing in microchannels. Lab on a Chip. (2013) 13:1121–32. doi: 10.1039/c2lc41248a 23353899

[14] Shields IV, Reyes CD, López GP. Microfluidic cell sorting: a review of the advances in the separation of cells from debulking to rare cell isolation. Lab on a Chip. (2015) 15:1230–49. doi: 10.1039/C4LC01246A 25598308 PMC4331226

[15] Wiklund M, Green R, Ohlin M. Acoustofluidics 14: applications of acoustic streaming in microfluidic devices. Lab on a Chip. (2012) 12:2438–51. doi: 10.1039/c2lc40203c 22688253

[16] Wang Y, Sayyadi N, Zheng X, Woods TA, Leif RC, Shi B, Time-resolved microfluidic flow cytometer for decoding luminescence lifetimes in the microsecond region. Lab on a Chip. (2020) 20:655–64. doi: 10.1039/C9LC00895K 31934716

